# Spatial Attention in Visual Working Memory Strengthens Feature-Location Binding

**DOI:** 10.3390/vision7040079

**Published:** 2023-12-18

**Authors:** Juyeon Joe, Min-Shik Kim

**Affiliations:** Department of Psychology, Yonsei University, Seoul 03722, Republic of Korea; juy1317@yonsei.ac.kr

**Keywords:** feature binding, spatial attention, visual working memory

## Abstract

There is a debate about whether working memory (WM) representations are individual features or bound objects. While spatial attention is reported to play a significant role in feature binding, little is known about the role of spatial attention in WM. To address this gap, the current study required participants to maintain multiple items in their WM and employed a memory-driven attention capture paradigm. Spatial attention in WM was manipulated by presenting an exogenous cue at one of the locations that memory items had occupied. The effects of spatial attention on attention guidance in visual search (Experiment 1) and memory performance (Experiments 1 and 2) were explored. The results show that WM-driven attention guidance did not vary based on whether the search features came from the same object in WM; instead, it depended on the number of features, regardless of their source object. In memory tasks, the cued object outperformed the uncued object. Specifically, the test item was better rejected when the features were mis-bound in the cued location than in the uncued location. These findings suggest that memory-driven attention guidance is feature-based, and spatial attention in WM helps bind features into object structures based on location.

## 1. Introduction

In daily life, we perceive objects that consist of multiple features. It is commonly believed that the visual brain processes objects as separate feature dimensions, such as color, shape, or orientation [1,2,3]. Since the features are processed in different areas, it is challenging for the brain to bind them appropriately. A growing body of literature has investigated how the different features are integrated into a coherent object, which is called the “binding problem”. Importantly, binding occurs at different levels of the visual system, including low- and mid-levels. It is already known that shape and color come apart in recall and perceptual encoding unless each colored item is fully attended [4,5]. The current study focuses on shape-color binding among the several types of binding, addressing whether features that are disconnected from each other at encoding can be integrated in working memory (WM).

There has been some disagreement regarding whether representations in WM are individual features or unified objects. Some studies suggest that the units of WM are integrated objects. This object hypothesis is strongly supported by the object-based effect in WM that conjunctions of two or more dimensions can be retained as well as a single dimension [6]. However, the object-based effect in WM was not replicated in subsequent studies [7,8,9]. Furthermore, the object-based effect may reflect location-based benefits [10]. Wang et al. (2016) reported that memory performance for conjunctions dropped when the conjunctions appeared at a single location [11]. Additionally, if bindings between features are maintained in WM, memory accuracy for each feature should covary. However, memory accuracy for each feature of the object was independent from other feature [12].

WM is closely intertwined with attention [13,14,15,16], such that the representations held in WM capture attention [17,18,19]. Based on this memory-driven attention capture paradigm, Thayer et al. (2022) examined the impact of WM representations on attention guidance [20]. In their experiments, participants were presented with two colored shapes and instructed to remember the color and shape of these objects. While maintaining these items in WM, they searched for a target letter. One of the search items matched both the color and shape of the remembered object in the same-object match condition and the feature of one remembered object and the other feature of the other in the different-object match condition. If the units of WM were bound objects, there would be differences in attention guidance between the same-object match condition and the different-object match condition. However, there was no significant difference between these conditions. They concluded that the units of WM-based attention guidance are individual features rather than integrated objects. Furthermore, their findings supported an indirect-binding model that suggests individual features are maintained separately [21] and bound through a shared location [22,23]. Location has been thought of as an index that mediates other features connected to the location [24]. When different objects were presented sequentially at the same location, one of the objects was likely to be mistaken for the other object presented at that location [25].

If it is assumed that spatial attention, known to play a significant role in feature integration, is allocated more to a particular location, could this lead to a difference between the same-object match condition and the different-object match condition? Spatial attention has been thought to serve as the glue that binds the individual features of an object together [26]. When the location of a memory item was attended, the time needed to bind nonspatial features decreased [27]. Several studies reported that memory performance for conjunctive items became poor when attention was diverted [28] or disrupted [29]. However, these prior studies examined the role of attention in feature binding in the case of attention being disturbed; there is a need to investigate the role of spatial attention within WM more directly. If attention plays an essential role in integrating individual features into a coherent object unit, allocating more attention to a specific item in WM would bind the features more effectively and affect memory performance for conjunctive items.

To examine this hypothesis, we used a variation of Thayer et al.’s (2022) procedure [20]. In our procedure, we manipulated spatial attention by making one of the locations where memory samples were presented flicker. This manipulation could shift attention allocation more to a specific location in WM. Attention can be guided by an endogenous or exogenous cue. An endogenous cue is known to be top-down and voluntary attention orienting, while an exogenous cue is known to be bottom-up and involuntary attention orienting. For example, an arrow presented at fixation guides attention in an endogenous way, and sudden onset guides attention to the cued location in an exogenous way. It is known that the endogenous cue and the exogenous cue are independent, yielding different behavioral effects and partially distinct neural substrates. Especially, endogenously oriented attention does not seem effective for feature binding [30]. Therefore, we used an exogenous cue to guide attention effectively. We investigated the effect of additional attention on WM guidance and memory performance. A critical search item was generated using a combination of features of each object. In the cued-object condition, the search item had the features of the cued object. In the uncued-object condition, the features of the uncued object were presented as the search item and expected to exhibit a weaker binding effect than the cued-object condition. In the combined condition, the search item used the combination of one feature from the cued object and the other from the uncued object. If additional spatial attention in WM facilitates binding between nonspatial features, there would be a bound representation for the cued object, and the magnitude of attention guidance would vary depending on the conditions. However, if spatial attention strengthens the binding between the features and their respective locations, resulting in a weaker (not strengthened) binding between nonspatial features, there would be no difference in attention guidance between the conditions. Additionally, we predicted that the memory performance for the cued object would be better than for the uncued object due to the enhancing effect of additional attention on any form of feature binding in WM. This experimental design allows an examination of how the representations held in WM are maintained and how spatial attention within WM affects the structure of the representations.

## 2. Experiment 1

### 2.1. Material and Methods

#### 2.1.1. Participants

Fifty-five students (42 female, 13 male) from Yonsei University participated in the experiment for course credit. All participants had normal or corrected-to-normal vision and no knowledge of the experiment’s hypothesis or purpose. They signed prior consent forms approved by the Yonsei University Institutional Review Board. The sample size was determined through a power analysis, using effect sizes obtained from a prior study [20]. The previous study set an effect size as $\eta_{\rho }^{2}$ = 0.18 to detect a medium-sized effect, and a power analysis using this effect size indicated that a minimum of forty-eight participants were required to obtain 80% power. Five participants were excluded from the analysis for correctly responding to the memory task in less than 65% of all trials.

#### 2.1.2. Apparatus

Stimuli were presented on a 24-inch LED monitor with a 60 Hz refresh rate and 1920 *×* 1080 resolution. The experiment was programmed via the Psychopy program. The distance between the participant and the monitor was kept constant at 57 cm through a chin-and-forehead rest.

#### 2.1.3. Stimuli

All stimuli were presented on a gray background (RGB: 192, 192, 192) with a central fixation cross (0.67° diameter). The memory and search objects were each a conjunction of color and shape. We chose five highly discriminable colors and five shapes. The five colors were yellow (230, 219, 38), red (225, 20, 36), green (44, 234, 25), fuchsia (224, 0, 233), and cyan (62, 230, 229). The five shapes were circle, triangle, diamond, pentagon, and hexagon. In the memory sample display, two colored shapes (each 6.29° × 6.29°) and two placeholders (each 8.36° × 8.36°) were centered 6.29° to the left and right of screen center.

#### 2.1.4. Procedure

On each trial, the color and shape values were chosen randomly without repetition from the five alternatives on each dimension. In the memory sample, one of the placeholders became bolder and flickered to guide spatial attention to that memory item (the cued item). For the search task, the search array consisted of two object items (each 6.29° × 6.29°). The possible locations in the search task were randomly selected from a set of six locations placed equidistantly along an imagery circle with a radius of 8.36° centered at fixation. In the neutral condition, the two objects were randomly constructed from the three colors and the three shapes not used in the memory sample display, resulting in no matches to the remembered feature values. Except for the neutral condition, there were eight search conditions. In each of the eight search conditions, one of the search items shared at least one feature with one of the memory items, while the other search item was a novel combination of features not used in the memory array. 

**Figure 1:**
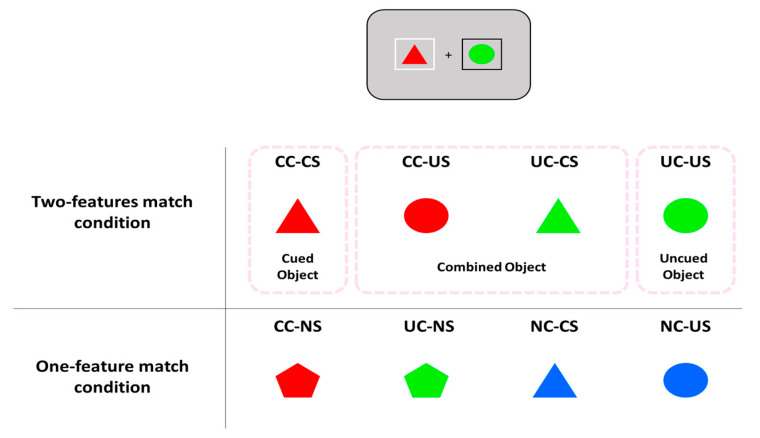
Search conditions of Experiment 1. The items (top) represent memory items, with the left item denoted by a white box, indicating the cued item. In the table at the bottom of the figure, the items could contain the search target (valid condition) or not (invalid condition). In the two-features match condition, one of the search items could match both the color and shape of the cued memory item (CC-CS), one feature of the cued item and the other feature of the uncued item (CC-US, UC-CS), or both the color and shape of the uncued memory item (UC-US). In the one-feature match condition, the search item could match only one feature (e.g., color or shape) of the cued item (CC-NS, NC-CS) or the uncued item (UC-NS, NC-US). The trials in the NC-NS condition correspond to invalid conditions.

The specific combination of features that matched those from the memory items varied depending on whether one or two features of one of the search items could match those from the memory items. In the two-features match condition, one of the search items could match both the color and shape of the cued memory item (Cued color-Cued shape; CC-CS), the color of the cued item and the shape of the uncued item (Cued color-Uncued shape; CC-US), the color of the uncued item and the shape of the cued item (Uncued color-Cued shape; UC-CS), or both the color and shape of the uncued item (Uncued color-Uncued shape; UC-US). In the one-feature match condition, one of the search items could match only the color of the cued item (Cued color-New shape; CC-NS) or the uncued item (Uncued color-New shape; UC-NS) or the shape from the cued item (New color-Cued shape; NC-CS) or the uncued item (New color-Uncued shape; NC-US). To further investigate the effects of WM representation structure, we collapsed CS-UC and US-CC conditions as a combined condition during data analysis (Figure 1). In other words, the combined condition indicated the recombination of the features across objects. 

The lines (1.27° × 2.1°) that appeared on the search objects were presented in the center of each object. One of the lines was randomly tilted 5° to the left or right. When there was a search object that matched features in memory, the object could contain the target line (valid condition) or not (invalid condition). In the memory test at the end of the trial, a test object was presented at the location where the matching feature had been located in the memory array. The test object either retained its original color and shape of the memory object at that location (same response), or one of its features changed to a different feature not exposed during the trial (different response). Note that the item to be tested was not relevant to the cue, and the location to be cued was counterbalanced. Participants were told that the cue was not relevant to the tasks.

**Figure 2:**
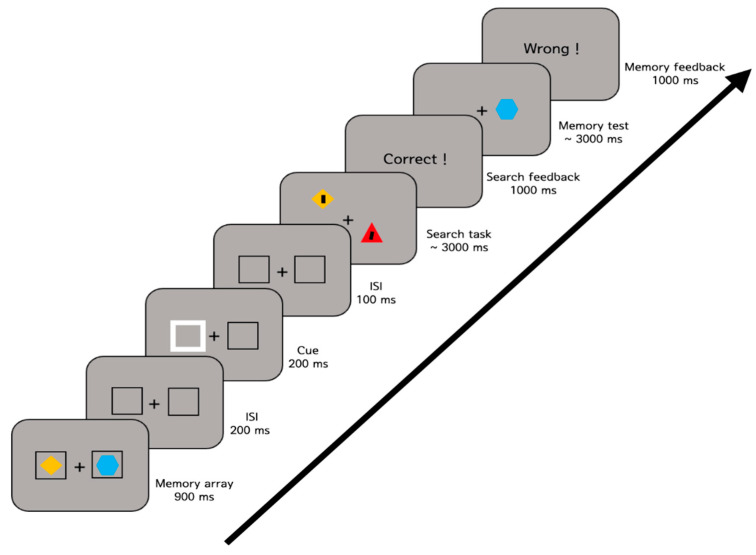
Sample trial sequence of Experiment 1. Participants were presented with two colored shapes (e.g., a yellow diamond and a blue hexagon). While participants memorized the objects, they searched a tilted line (search target). After the search task, they responded to indicate whether the test item (e.g., a blue hexagon) was the same or different than the memory object at that location.

The experimental procedure is illustrated in Figure 2. Each trial began with a fixation cross presented for 500 ms, followed by the memory array for 900 ms. After a 200 ms delay, one of the placeholders flickered for 200 ms and returned to its original appearance for 100 ms. After the placeholders disappeared, the search array was presented for up to 3000 ms, and then the memory test was presented for up to 3000 ms. For the search task, participants pressed the left or right arrow key to indicate the orientation of the tilted line. For the memory test, they used the “s” (same) or “d” (different) key to indicate whether the test object was the same or different than the memory object at that location. Participants were encouraged to respond as fast as possible while still being accurate. Feedback was provided for both tasks to encourage participants to concentrate on the task.

Participants first completed a practice block of 12 trials. They then completed three blocks of experiment trials. Each block contained 144 trials. In each block, trials were evenly split between valid and invalid conditions, except for the neutral condition. Participants completed a total of 432 experimental trials. The entire experiment lasted approximately 40 min.

### 2.2. Results

#### 2.2.1. Search Task

We used only trials with both correct search and memory responses in the analysis of visual search response times (RTs) and memory accuracy. RTs faster than 200 ms on the search task and trials with an RT that was 3 standard deviations (*SD*) above or below the mean for each participant were excluded from the analyses. An average of 1.30% (*SD* = 0.51%) of trials per participant were removed by applying these exclusion criteria.

**Figure 3:**
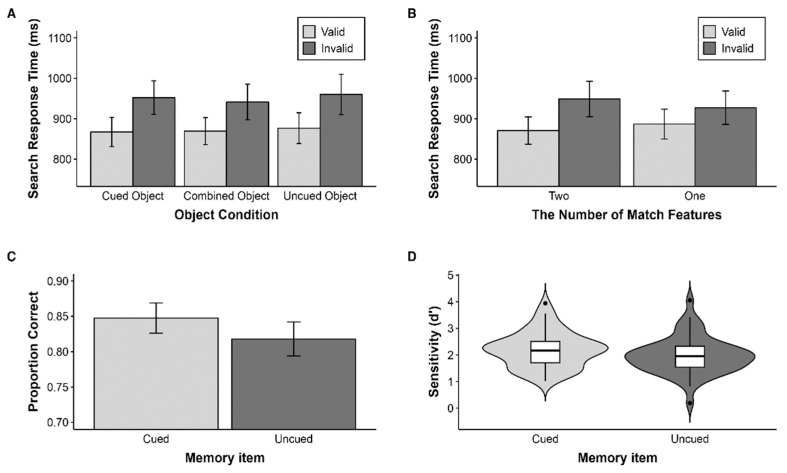
Results of Experiment 1. Error bars represent 95% confidence intervals. (**A**) Mean response times (RT) in the search task for Experiment 1 as a function of object condition and validity. (**B**) Mean RT in the search task for Experiment 1 as a function of number condition and validity. (**C**) Proportion of correct responses and (**D**) sensitivity in the memory task for Experiment 1.

To investigate the impact of WM representation structure on WM-driven attention guidance, particularly when spatial attention was directed by the cue, we compared RTs in the two-features match condition. Mean RTs from the conditions were submitted to a 3 (Object condition: Cued object/Combined object/Uncued object) *×* 2 (Search validity: Valid/Invalid) repeated measures analysis of variance (ANOVA). *p* values were adjusted using the Greenhouse–Geisser epsilon correction used for nonsphericity. There was no significant effect of object condition, *F*(2, 98) = 1.95, *p* > 0.05. The main effect of search validity was significant, *F*(1, 49) = 63.82, *p* < 0.001, $\eta_{\rho }^{2}$ = 0.57, with faster RTs in the valid condition (*M* = 879.86, *SD* = 241.00) than the invalid condition (*M* = 933.11, *SD* = 255.82). There was no significant interaction between object condition and search validity, *F*(2, 98) = 0.61, *p* > 0.05 (Figure 3A). These results indicate that memory-driven attention guidance did not significantly differ based on whether the features came from the same object or not. To examine the effect of the cue, all search conditions were categorized according to whether the features held in WM were cued when they appeared in the search task. The cued condition involved CS-CC, CS-NC, and NS-CC; the uncued condition involved US-UC, US-NC, and NS-UC. The combined conditions (i.e., CS-UC and US-CC) were excluded because the cued feature and uncued feature were mixed. A two-way ANOVA with cue type revealed that there was no main effect of cue, *F*(1, 49) = 1.37, *p* > 0.05. There was only a main effect for search validity, *F*(1, 49) = 51.15, *p* < 0.001, $\eta_{\rho }^{2}$ = 0.51, with faster RTs in the valid condition (*M* = 881.74, *SD* = 128.23) than the invalid condition (*M* = 936.94, *SD* = 148.83). There was no significant interaction between cue condition and search validity, *F*(1, 49) = 2.30, *p* > 0.05. These results suggest that the cue does not modulate attention guidance from WM. 

To investigate whether WM-driven attention guidance varies depending on the number of features held in WM that were presented during the search task, we compared the validity effect of the two-features match condition and the one-feature match condition (Figure 3B). We conducted a 2 (the number of features: two/one) *×* 2 (search validity: valid/invalid) repeated measures ANOVA. There was no main effect of the number of features, *F*(1, 49) = 0.49, *p* > 0.05. There was a main effect of search validity, *F*(1, 49) = 56.64, *p* < 0.001, $\eta_{\rho }^{2}$ = 0.54, with faster RTs in the valid condition (*M* = 877.38, *SD* = 239.76) than the invalid condition (*M* = 937.32, *SD* = 256.89). Critically, there was a significant interaction between the number of features and search validity, *F*(1, 49) = 33.91, *p* < 0.001, $\eta_{\rho }^{2}$ = 0.41. The validity effect was larger in the two-features match condition than the one-feature match condition. These results support that feature is a fundamental factor in attention guidance from WM.

#### 2.2.2. Memory Task

The average accuracy for each condition (i.e., cued object and uncued object) was calculated. A one-way repeated measures ANOVA with the factor of memory test object condition (cued vs. uncued) revealed a main effect of memory condition, *F*(1, 49) = 17.36, *p* < 0.001, $\eta_{\rho }^{2}$ = 0.26 (Figure 3C). Participants remembered the cued object (*M* = 0.85, *SD* = 0.08) better than the uncued object (*M* = 0.82, *SD* = 0.09). We next computed memory sensitivity *d’* = z(hit rate) − z(false alarm rate) according to signal detection theory [31]. Participants had better memory sensitivity for the cued object compared to the uncued object, *F*(1, 49) = 17.00, *p* < 0.001, $\eta_{\rho }^{2}$ = 0.26 (Figure 3D). These results suggest that directing more spatial attention to a specific item in WM affects memory accuracy even though the cue was irrelevant to the memory task.

### 2.3. Discussion

In Experiment 1, we asked whether allocating spatial attention to a certain item in WM could affect the structure of WM representation. After the memory sample array disappeared, we manipulated spatial attention by presenting a cue to a certain location. No significant difference was observed in WM-driven attention guidance during the search task among the cued object condition, the uncued object condition, and the combined object condition. This result is consistent with Thayer et al.’s (2022) finding that visual working memory (VWM)-driven attention is not affected by whether the matching features came from the same object [20]. Instead, we found an effect of the number of matching features on WM-driven attention guidance: the more the features of VWM representations matched the search item, the larger the attention guidance was. These results indicate that WM-driven attention guidance is feature-based.

In the memory task, the cued objects were remembered better than the uncued objects, suggesting that directing spatial attention to a certain item in WM improves memory performance even though the cue was irrelevant to the memory task. It is implausible that directing spatial attention to a specific item in WM facilitates the binding of features into a coherent object. Instead, spatial attention in WM plays a role in enhancing memory accuracy.

## 3. Experiment 2

To elucidate the effect of spatial attention on memory performance, we conducted only a memory task in Experiment 2. Spatial attention was manipulated through an exogenous cue directing attention to a specific item within WM, as in Experiment 1. The memory array consisted of two uncued items and one cued item. This manipulation allowed us to compare cued features with uncued features. If spatial attention strongly facilitates the binding between nonspatial features, such as color and shape, the cued location would have a bound representation with no potential for confusion among any feature combinations. Alternatively, if spatial attention enhances each feature representation at its location or facilitates the binding between each feature and its respective location, the recognition performance would depend on the individual feature level. In other words, there would be a difference between the combinations because of strengthened feature-location binding. The memory performance would be high in the case of a completely new object since there would be no features held in WM.

### 3.1. Material and Methods

#### 3.1.1. Participants

The sample size of Experiment 2 was determined based on Experiment 1. The power analysis revealed that a sample size of *N* = 48 was required to achieve the desired effect size, so 49 new participants were recruited for Experiment 2. Data from one participant were excluded from the analysis for correctly responding to the memory task in less than 65% of all trials.

#### 3.1.2. Apparatus, Stimulus, and Procedure

**Figure 4:**
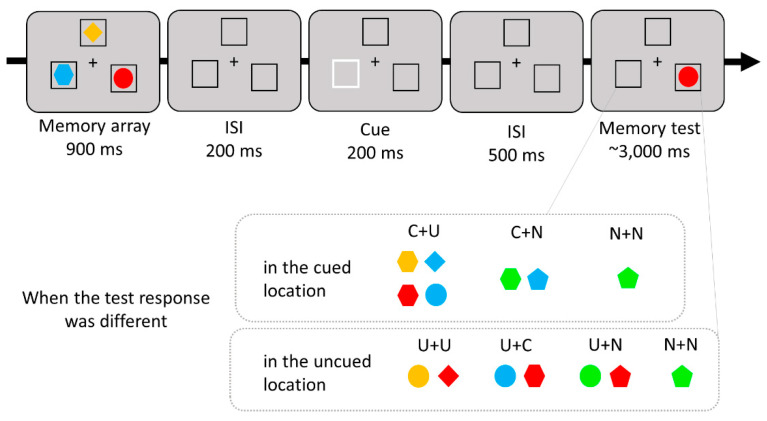
Sample trial sequence and conditions of Experiment 2. When a different item appeared in the cued location, the test item could match one feature of the cued object and the other of the uncued object (C+U), one feature of the cued object and the other of the new object (C+N), or both two features of the new object (N+N). In the uncued location, one feature of the test item could match one of the original features associated with that location and the other feature could match either the shape or color of another uncued item (U+U), a cued object (U+C), or a new object (U+N), or the test item could match both features of the new object (N+N). Except for the N+N condition, each location contained at least one original feature associated with that location.

The stimuli and procedure were the same as in Experiment 1, except there were three memory items and no search task. In Experiment 2, three colored shapes and three placeholders were presented at fixed locations, equally distributed around the circle (arc length of 60° between stimuli centers). After the memory array disappeared, one of the three placeholders became bolder and flickered to guide spatial attention. Therefore, there were one cued item and two uncued items in VWM. In the memory test array, one of the items was presented, and it either retained its original color and shape (same response), or at least one of its features changed to a different feature (different response). In this study, we manipulated various cases in which the test item differed to examine the comparative effects of cued and uncued features within WM on the recognition process, in contrast to novel features not stored within WM. When a different item was presented in the cued location, either the shape or color of the item could match one of the uncued objects (Cued feature-Uncued feature; C+U), a new object (Cued feature-New feature; C+N), or both the shape and color of the item came from a new object (New feature-New feature; N+N). In the case of the uncued location, either the shape or color of the item could match another uncued item (Uncued feature at that location-Uncued feature at other location; U+U), the cued item (Uncued feature at that location-Cued feature; U+C), a new object (Uncued feature at that location-New feature; U+N), or both the shape and color could come from a new object (New feature-New feature; N+N). Other than in the N+N condition, each location in the memory test contained at least one original feature associated with that location. The experimental procedure and test conditions are illustrated in Figure 4.

Each trial began with a fixation cross presented for 500 ms, and then the memory array was presented for 900 ms and then disappeared. After 200 ms, one of the placeholders became bolder for 200 ms. After a 500 ms delay, the memory test item appeared. Participants were instructed to press the “s” (same) or “d” (different) key to indicate whether the test item at that location matched the memory item at that location. They were encouraged to respond as fast and accurately as possible. Feedback was provided to encourage participants to concentrate on the task. The main task was preceded by 12 practice trials and comprised 504 trials divided into four blocks. The entire experiment lasted approximately 40 min.

Participants had to remember three colored shapes, and the method to manipulate spatial attention was the same as in Experiment 1.

### 3.2. Results

**Figure 5:**
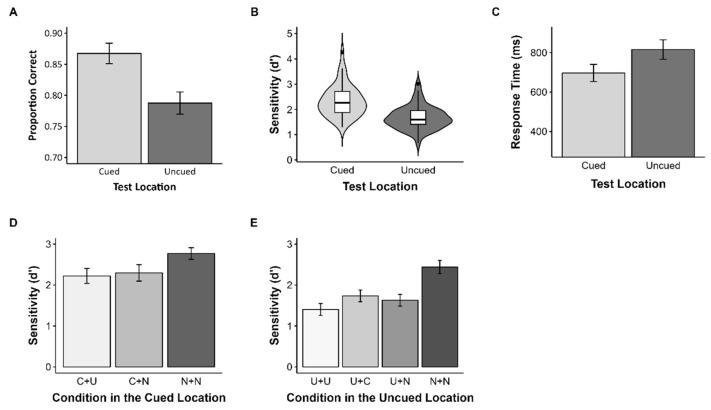
Results of Experiment 2. Error bars represent 95% confidence intervals. (**A**) Proportion of correct answers, (**B**) sensitivity, (**C**) mean RT in Experiment 2, (**D**) sensitivity in the cued location, and (**E**) the uncued location when the test item differed from the memory item.

To investigate the effect of the cue on WM representation, we conducted a one-way repeated measures ANOVA. Like Experiment 1, participants remembered stimuli in the cued location (*M* = 0.87, *SD* = 0.06) better than the uncued location (*M* = 0.79, *SD* = 0.06), *F*(1, 47) = 141.14, *p* < 0.001, $\eta_{\rho }^{2}$ = 0.75 (Figure 5A), and showed higher sensitivity for the cued location (*M* = 2.33, *SD* = 0.62) compared to the uncued location (*M* = 1.67, *SD* = 0.46), *F*(1, 47) = 94.29, *p* < 0.001, $\eta_{\rho }^{2}$ = 0.67 (Figure 5B). Moreover, the reaction time for objects in the cued location (*M* = 696.62, *SD* = 150.09) was significantly faster than for those in the uncued locations (*M* = 815.57, *SD* = 160.97), *F*(1, 47) = 169.62, *p* < 0.001, $\eta_{\rho }^{2}$ = 0.78 (Figure 5C).

To further elucidate the effect of the cue, we analyzed the different cases at each location. First, in the cued location, there was a significant main effect of the test condition, *F*(2, 94) = 42.17, *p* < 0.001, $\eta_{\rho }^{2}$ = 0.47 (Figure 5D). The sensitivity for the N+N condition (*M* = 2.77, *SD* = 0.49) was significantly higher than for the C+U condition (*M* = 2.22, *SD* = 0.63) and C+N condition (*M* = 2.30, *SD* = 0.69). There was no significant difference between the C+U condition and the C+N condition. There was also a significant main effect of the test condition in the uncued location, *F*(3, 141) = 119.93, *p* < 0.001, $\eta_{\rho }^{2}$ = 0.72 (Figure 5E). Like the cued location, the sensitivity was highest in the N+N condition (*M* = 2.44, *SD* = 0.56) and lowest in the U+U condition (*M* = 1.40, *SD* = 0.50). The sensitivity in the U+C condition (*M* = 1.74, *SD* = 0.49) and the U+N condition (*M* = 1.63, *SD* = 0.50) was higher than the U+U condition, but the difference between the U+C condition and the U+N condition was not significant.

### 3.3. Discussion

In Experiment 2, we examined the effect of spatial attention in WM on feature binding. Three colored shapes were presented and the location of one of them was cued once the shapes had disappeared from the screen. Note that the cue was uninformative and task-irrelevant. The role of the cue was to allocate additional spatial attention to a specific item within WM. Participants responded faster when asked to respond to the cued location and memorized the cued items better than the uncued items. This result demonstrates that allocating additional spatial attention in WM affects memory performance.

Since participants were not required to perform an articulatory suppression task, there is a possibility that verbal strategy could be involved in memory performance. Verbal strategy has been effective in improving memory performance [32]. However, a recent study using a change detection paradigm found that verbalization did not contribute to VWM performance in younger adults [33]. Moreover, the strategy was more demanding for conjunctions of features compared to single features [34]. Therefore, it is unlikely that additional attention improves VWM performance by facilitating verbal rehearsal.

Specific conditions were designed to explore whether spatial attention facilitates the binding of nonspatial features or the binding of each nonspatial feature with its corresponding location. In the cued location, the sensitivity for the C+U condition was not significantly different from the C+N condition. The sensitivity was the highest in the N+N condition. If spatial attention in WM strongly bound the nonspatial features to an object, there would be no significant differences among the conditions, regardless of the feature combinations presented. In other words, if there was a robust bound representation consisting of nonspatial features, an object comprising both cued and uncued features should be treated as a completely new object, even when the cued feature within the item is present. Therefore, the results in the cued location suggest that additional spatial attention enhances the relationship between a feature and its corresponding location. Consistent with the indirect binding model, which suggests that features of the same object are bound indirectly through the object’s location, in this study, spatial attention seemed to strengthen the feature-location binding rather than the binding between nonspatial features. The results in the uncued location seem to align with those in the cued location. The sensitivity was the lowest in the U+U condition. Since spatial attention did not facilitate binding between the uncued features and their locations, there would be a higher possibility that participants would misrecognize the different items consisting of the uncued features. The difference between the U+C and U+N conditions was not significant, and the sensitivity was the highest in the N+N condition. These results suggest that spatial attention enhances the binding of nonspatial features to specific locations, and that the cued feature has a special index only in that location. If the cued feature appeared in other locations, such as an uncued location, the feature seemed to be treated as a new feature even though the feature was held in WM. Furthermore, the results that showed that WM performance in the N+N condition was the highest in both the cued and uncued locations suggest that memory performance could depend on individual features [35], especially the relationship between individual features and their corresponding locations. These findings indicate that spatial attention in WM, manipulated through uninformative exogenous retro-cues, facilitates feature-location bindings, which is consistent with previous research showing that retro-cues result in stronger item-context bindings [36]. 

## 4. General Discussion

This study investigated the role of spatial attention in feature binding. In Experiment 1, there was no significant difference in attention guidance across the cued-object condition, the uncued-object condition, and the combined-object condition. This result shows that WM-driven attention guidance is based on individual features. In Experiment 2, there was a difference in response times between the cued and uncued locations, and the memory accuracy of the cued location was higher than that of the uncued location. In the cued location, there was no difference between the C-U and C-N conditions, while the sensitivity for the U-U condition was lower than the U-C and U-N conditions in the uncued location. If spatial attention facilitates the binding of an object’s nonspatial features directly, the cued location would have a bound representation, and there would be no difference between any form of combinations, including the N-N condition. Therefore, additional spatial attention in WM seems to facilitate the binding between the nonspatial features of an object and its location rather than enhancing the binding between nonspatial features. Moreover, there was no difference between the U-C and U-N conditions in the uncued location, which suggests that the cued feature gets a special index in that location, potentially being treated as a new feature when presented elsewhere. Overall, the results of the current study align with previous results that support an indirect binding model [20,21,22,23], even though we manipulated spatial attention.

Attention has been thought to actively interact and be closely intertwined with WM [37,38,39,40,41,42,43,44,45]. However, the current study indicates that object structure in WM does not influence attention. Whether the features belonged to the same object or not, attention was driven by individual features. In contrast with the current study, Hollingworth and Beck (2016) suggest that VWM-based attention capture is driven partially by higher-level representations that integrate information from multiple items in VWM [46]. In their experiments, participants memorized two-colored items and then searched for a target. The search array contained either no or two-colored distractors. When there were two-colored distractors, the number of colors matching the items in VWM were either zero (match-0), one (match-1), or two (match-2). The capture effect for match-1 was not significantly different from the capture effect for match-0, and the capture effect for match-1 was found to be less pronounced than the capture effect for match-2. This result reflects partial feature repetition and suggests the possibility of storing items in VWM as part of composite representations. However, there is a critical difference between the present study and Hollingworth and Beck’s experiment. We used combinations of two different feature dimensions (shape and color) as a proxy to manipulate attention guidance, while Hollingworth and Beck used separate features in one feature dimension (color). There would be competition between features in the same dimension for limited resources in VWM [47,48]. Therefore, competition between each feature in the same dimension might weaken resolution and result in an overall underadditive capture effect. This finding aligns with previous research that demonstrated that there was no object-based effect of memory performance for objects consisting of the same feature dimension (e.g., color-color conjunction), but there was an effect for different feature dimensions [9]. 

It is also informative that eye movement may serve as a factor that affects feature binding. The utilization of the exogenous cue in the present study induced shifts in eye movement. There has been a substantial overlap between VWM, spatial attention, and the oculomotor system [49,50]. Focusing attention on a specific location facilitated transferring that location into VWM [51,52], and VWM was disturbed by an abrupt eye movement [53]. Furthermore, the types of error in VWM appear to be contingent upon the processing of eye movement. The probability of misreporting non-target colors was high when attention shifted from one location to another location. In contrast, in the case of attention splitting, the probability of blending in feature space between two attended locations was high [54]. Therefore, eye movement can be a crucial factor in feature binding [55]. Further work is required to examine the mechanism by which dynamic remapping through eye movements affects feature binding in VWM.

The results of the memory tasks indicate that the features of the object are not bound directly to each other but rather bound through locations [23]. Location has been shown to be an important factor in working memory [10,11,56]. Moreover, the retro-cue method, which we used to manipulate spatial attention, is assumed to affect memory performance by strengthening item-context (e.g., spatial location) binding [36,57]. Therefore, the memory enhancement observed in the current study could be due to the strengthened bindings between individual features and their locations. Furthermore, the results in the uncued location suggest that the cued feature could get a special index in that location. This result is in line with the object file [58,59], which suggests that the features of an object are bound to its location, which is called the spatial index, when the objects are attended. This spatial index forms the object file containing the features of the object to maintain a continuous representation of the object. Therefore, in the present experiments, the spatial index formed by the retro-cue might form an object file and isolate the features at the cued location from those at the uncued locations. This index is ecologically advantageous as we process and categorize numerous stimuli in our daily lives. For instance, when we stop at an intersection and wait for the traffic light to change, we do not confuse the green color of the trees or anything else in the surroundings with the green light. Feature binding through strengthening the binding between the features and their location proves to be a valuable strategy for enhancing memory performance by indexing the features.

However, it is important to explore whether each feature dimension has a symmetrical binding effect. Color is assumed to have a relatively large priority in the top-down attention guidance over other dimensions, such as orientation and location [42,60,61]. Moreover, Rajsic and Wilson (2014) found asymmetrical access to color and location in VWM, and the effectiveness of retrieving location with a color cue surpassed that of retrieving color with a location cue [62]. Therefore, it is not certain whether the binding effect through additional attention resulted from an overall enhancement in each feature-location binding or a specific enhancement in a particular feature representation. Since the current study focused on the relationship between attention and the combination of features, additional work on how attention affects each feature dimension would contribute to a better understanding of the binding effect through attention.

Retention time could impact VWM representation. The magnitude of the binding effect could differ, especially if there were variations in the retention duration. The prior studies suggest that consolidation is a rapid process, making it possible to form about three VWM representations with a delay of 100–200 ms after memory-array offset [63,64,65]. However, van den Ven et al. (2012) found that presenting a mask at 200 ms stimuli offset disturbed WM performance [66]. Moreover, the functional relevance of the early visual cortex to VWM has been supported [67]. Therefore, there is a possibility that additional attention presented at 200 ms memory-array offset could facilitate the consolidation of the cued object, resulting in strengthened binding.

## 5. Conclusions

This study examined the role of spatial attention in WM and found that attention guidance from WM is based on individual features and unaffected by the structure of WM representation, whether the features belong to the same object or not. This study highlights the mechanism of spatial attention in affecting feature binding that strengthens the feature-location binding. It suggests that feature binding, by indexing features in specific locations, is a beneficial strategy to protect and enhance memory performance. Nevertheless, further research is needed to explore to what extent and how, if at all, additional attention would influence nonspatial feature binding.

## Data Availability

The datasets for this study can be found in the https://osf.io/xt8sy/?view_only=688ec4b0cdc044c2b7d8ee3be972e073 OSF repository (accessed on 17 December 2023).
